# Inpatient Physical Therapy in Moderate to Severe Traumatic Brain Injury in in Older Adults: A Scoping Review

**DOI:** 10.3390/ijerph20043367

**Published:** 2023-02-14

**Authors:** Florence Noël, Marie-Pier Gagnon, Jasmine Lajoie, Marjorie Côté, Sarah-Maude Caron, Abygaël Martin, Alexis Labrie-Pichette, Livia P. Carvalho

**Affiliations:** 1 Faculty of Medicine and Health Sciences, School of Physical and Occupational Therapy, University of Sherbrooke, Sherbrooke, QC J1H 5N4, Canada; 2 Aging Research Center, Centre de Recherche sur le Vieillissement du CIUSSS de l’Estrie-CHUS, Sherbrooke, QC J1H 4C4, Canada

**Keywords:** physiotherapy, TBI, acquired brain injury, aged, elderly, hospital, acute care

## Abstract

Background: Around 500/100,000 Canadians experience a traumatic brain injury (TBI) resulting in long-term disabilities and premature death. Physiotherapy is known to positively impact the prognosis of young adults following a TBI. Objective: This is a scoping review that aimed to identify research topics in physiotherapy interventions for seniors after a TBI, describe potential knowledge gaps, and uncover needs for future research. Methodology: Ten databases were interrogated (January–March 2022). We included texts published after 2010, in English or French, scientific papers, guidelines, and gray literature sources targeting in-hospital, acute-to-subacute interventions for people aged ≥55 years old with a moderate-to-severe TBI. The outcomes sought were physical/functional capacities, injury severity, and quality of life. Results: From 1296 articles, 16 were selected. The number of participants from the studies altogether was 248,794. We identified eight retrospectives studies, three clinical trials, and five articles from the gray literature. Articles were classified according to the nature of their analysis and outcomes: (1) interventional studies including physiotherapy (at least 10 types of rehabilitative or preventive interventions were identified); (2) studies evaluating prognostic factors (five factors identified); and (3) recommendations from clinical practical guidelines and other sources (gray literature). Our results provide evidence that physiotherapy is effective in TBI acute rehabilitation for the elderly to prevent complications arising from the primary injury and to improve functional capacities. Conclusion: The heterogeneity of our results does not allow us to infer the effectiveness of one intervention over another. However, we found that the elderly population benefits from physiotherapy interventions as much as adults, but the gap must be filled with higher-quality studies to make definite recommendations.

## 1. Introduction

Globally, it is estimated that 69 million people will suffer from a brain injury in their lifetime [1]. In Canada, it is estimated that 500/100,000 people will suffer from a traumatic brain injury (TBI) every year [2]. In the province of Quebec, more specifically, moderate-to-severe TBI cases lead most of the time to hospitalization and are associated with the highest death rate among all diagnosed trauma (18.2% based on data collected from 2013 to 2016) [3]. Most often, the causes of TBI are related to a head impact caused by car accidents in younger and falls in older adults [4]. In 2021, seniors represented 18.5% of the total population of Canada [5] and this proportion tends to increase, reaching up to 23% in 2030 according to estimates [6]. Knowing that approximately one-third of elderly people living in the community will have an episode of fall in one year, the population aging phenomenon will certainly continue to contribute to the increase in the prevalence of TBI over time in this clientele [7]. It has been reported that falls cause 85% of injury-related hospitalization among older adults [8]. Moderate-to-severe TBI differs from a non-traumatic head injury and a mild traumatic brain injury both in terms of treatment and typical evolution and prognosis [9], with the severity—usually classified according to the Glasgow Coma Scale (GCS) at hospital admission [9]—not only being correlated with the level of tissue damage [10] but also with long-term levels of disability, morbidity, and healthcare services needs and utilization [11].

Despite the inpatient rehabilitation phase being critical in influencing the long-term prognosis, there is very limited evidence in terms of potential interventions for older adults during this period. Indeed, according to the National Institute of Excellence in Health and Social Services of Quebec, which published a clinical practice guide on adults who have suffered a TBI [3], a separate literature review process would be necessary to allow the creation of a clinical practice guide including specific recommendations to the elderly, since the evidence related to this age group is much more limited. Also, the typical profile of the elderly, associated with the complexity of the coexistence of other health conditions and the issue of polypharmacy, makes treatment sometimes difficult and challenging, but greatly necessary [12]. Given the increase in the number of comorbidities with age, older adults are at greater risk of mortality in hospital settings [12] and of having a slower recovery and being less functional at the time of hospital discharge [13]. Physiotherapy (PT), known to be effective in minimizing long-term physical and functional consequences in many acute conditions, has an important role to play in these contexts [14,15].

The objective of this scoping review is therefore to examine the literature to (1) identify research topics in acute-care physical therapy interventions for older adults after a TBI and the measures used in terms of quality of life, function, and severity outcomes, (2) describe potential knowledge gaps in this topic, and (3) uncover further needs for future research. Our results will hopefully contribute knowledge and evidence-based practice on what can be achieved by physical therapists with older patients after a TBI in real life and inform future research in the field.

## 2. Methods

The scoping review procedure adhered to the PRISMA-ScR methodological framework for scoping reviews [16]. Specifically, the procedure was as follows: (1) identification of the research question, which was: *What is the current evidence on acute or subacute physical therapy rehabilitation to treat older people who have undergone a moderate or severe TBI?* (2) literature search to find relevant studies, (3) selection of studies pertaining to our research question, (4) extraction of data, and (5) grouping, summarization, and reporting of results.

### 2.1. Inclusion Criteria

We included all documents meeting the following inclusion criteria: (1) randomized controlled trials (RCTs), studies including one or more clinical recommendations for the targeted population, non-randomized or mixed-method clinical studies including interventions in physiotherapy, (2) studies including patients with a moderate or severe brain injury, aged 55 or over (studies evaluating younger age groups were included only if an older age group was evaluated separately, as a subgroup), (3) studies including physical and functional outcomes (e.g., walking, going up and down stairs, ability to perform activities of daily living, mobility, muscle strength and/or power, aerobic or cardiorespiratory capacity, endurance, balance, postural control, motor control), severity level (GCS or Glasgow outcome scale at follow up assessment visits [17]) and quality of life (any specific or non-specific measure of health-related quality of life), (4) studies published in French or English, and, finally, (5) studies published between 2010 and 2022.

Narrative, rapid, and mini-reviews, qualitative research, studies including only rehabilitation interventions other than physiotherapy, interventions carried out in an outpatient setting (ambulatory clinics, community, or long-term care centers) or including people with mild traumatic brain injury, were excluded.

### 2.2. Information Sources

From January to March 2022, searches of 10 online databases (Medline, CINAHL, AMED, Angeline, Social abstract in gerontology, PEDRO, Pubmed, SportDiscus, Cochrane, and Scopus) were performed to find the studies that were relevant to our research question, including texts published after 2010 and before June 2022 written in English or French.

### 2.3. Search Strategy

A librarian with expertise in different types of reviews in health sciences assisted in the process of developing search strategies and determining relevant databases accordingly. Because very few studies were selected in this first stage, we carried out a second search stage that consisted of searching for articles from gray literature sources. The complete search strategy for one of our databases (Academic Search Complete—EBSCOhost) was based on the following terms: (Brain injur* OR ‘‘Traumatic brain injury’’ OR ‘‘Traumatic N3 Brain injur*’’ OR ‘‘Traumatic brain injur*’’ OR ‘‘Head injur*’’ OR ‘‘Crushing brain injur*’’ OR ‘‘Crushing skull injur*’’ OR ‘‘Craniocerebral injur*’’) AND (Elder* OR Older OR Geriatri* OR Senior* OR Aging) AND (Rehabilitat* OR Physiotherap* OR ‘‘Physical Therap*’’ OR Exercis*) AND (Hospit* OR Inpatient OR Acute OR Subacute) NOT (surgery OR operation OR ‘‘surgical procedure’’ OR ‘‘surgical treatment’’ OR operative OR postoperative OR post-operative) NOT (stroke OR ‘‘cerebrovascular accident’’ OR cva OR ‘‘cerebral vascular event’’ OR cve OR ‘‘transient ischaemic attack’’ OR tia) NOT (children or adolescents or youth or child or teenager) NOT (‘‘mild traumatic brain injury’’).

### 2.4. Selection of Sources of Evidence

We searched the literature targeting, specifically, in-hospital, acute-to-subacute, PT interventions (exclusively or combined with other types of interventions) delivered to older adults (55 or older) who have undergone a moderate or severe TBI and have been hospitalized. Randomized and non-randomized trials, guidelines, book chapters and conference abstracts were included, while narrative reviews, rapid or mini-reviews and qualitative studies were excluded.

### 2.5. Data Charting Process

Pairs of a six-member disciplinary team independently screened articles in two steps: titles and abstracts then full-text documents, after checking for duplicates. Disagreements were resolved through consensus between the original pair of reviewers and, when necessary, with the assistance of a third member. The Zotero software, a reference management system [18], was used for data selection, abstraction, duplicate analysis, and file storage and organization. The team developed a charting spreadsheet that was pilot tested using random articles together with the group to determine relevant items: e.g., year and country of publication, journal title, report type, and intervention characteristics (i.e., setting, intervention description, outcomes, results in summary).

After completing the final selection, we identified two distinct groups of articles: the first consisted of classic intervention studies, and the second, consisted of articles that investigated prognostic factors and/or studies addressing prognostic factors and/or responses resulting from inpatient rehabilitation (with no detailed description of the specific PT interventions).

### 2.6. Data Items

Variables or concepts for which data were sought were physical or functional capacities (aerobic, strength, mobility, etc.), TBI severity level, and quality of life, either as primary or secondary outcomes.

### 2.7. Critical Appraisal and Synthesis of Results according to Quality of the Selected Studies

To assess the quality of the articles, we used 2 different scales: 1) the Downs and Black checklist [19] which is suitable for quantitative study methodologies (randomized and non-randomized clinical trials) that evaluate five dimensions: how the results are reported, internal validity (bias and confounding), external validity, and power. The percentage of met criteria was reported based on the number of criteria/subcategories applicable to the study in question. A total score of 28 points could be assigned. A study rated as excellent has 26 to 28/28, a good quality study has 20 to 25/28, a study with a score of 15 to 19/28 is rated as acceptable, and a poor study has a score of ≤14. We analyzed retrospective studies separately since some criteria did not apply. For these, the scale had a maximum score of 19/19. This modified version rates as excellent a score of 18 to 19/19, as good a rating of 14 to 17/19, as acceptable a rating of 8 to 13/19, and scores under 8 are considered poor.

For the gray literature, we used the Authority, Accuracy, Coverage, Objectivity, Date and Significance (AACODS) checklist, which is an evaluation and critical appraisal tool specifically for use with gray literature sources. A score of ≤60% is considered poor, a score between 61 and 84% is considered acceptable, a score between 85 and 90% is good, and a score between 91 and 100% is considered excellent (scale based on a total of 32 points) [20]. Details of the way scores were assigned as well as their results can be found in Appendix A and Appendix B.

## 3. Results

**Figure 1:**
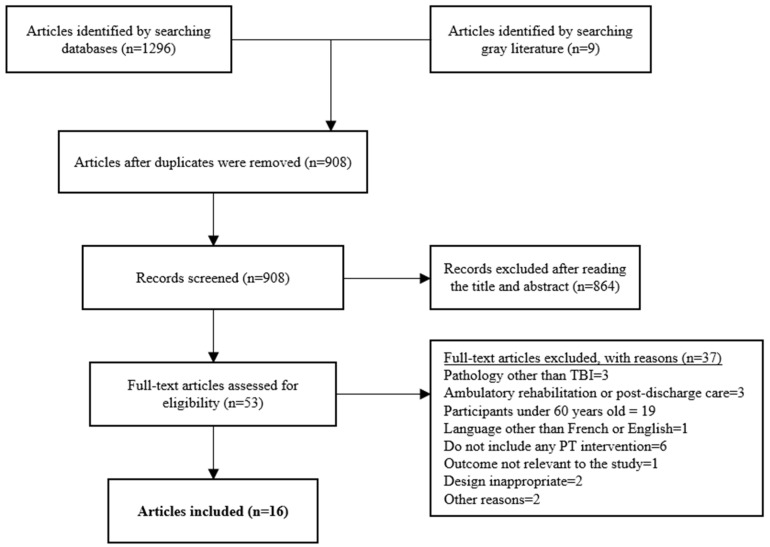
Flowchart according to PRISMA for Scoping Reviews.

We initially identified a total of 1296 articles. As mentioned above, we removed 388 articles in duplicate. After the first screening of abstracts and titles, 864 articles were excluded because they did not meet inclusion criteria. At this point, we added the gray literature search. A manual search (“snowball” strategy, consisting of searching for additional articles from the references mentioned in the articles already selected) was also attempted in order to ensure the identification of articles that would not have been covered by previous databases. However, this search was inconclusive. After this second-stage screening, we obtained a total of 44 scientific articles and 9 articles from the gray literature. After the final screening, 5 articles from the gray literature and 11 scientific studies were included, a total of 16 articles meeting all the inclusion criteria (Figure 1). The number of participants from the studies altogether was 248,794, which is excellent considering the small number of articles. We had eight retrospective studies, three clinical trials and five articles from the gray literature (Table 1).

### 3.1. Interventional Studies

The first category of articles was the classic interventional studies. In one study [21], the participants were divided into two groups. One group had stationary ergocycle sessions 5 x/week for 4 weeks in addition to the usual 1-h therapy, the latter including strength training, endurance, balance, coordination, and the practice of functional tasks. In addition, the participants had group classes 6 x/week. The second group received usual care only. The authors reported similar outcomes between the two groups. However, even without significant differences between the two groups, they found an important improvement in both. They were able to confirm that receiving rehabilitation, in general, promoted beneficial and measurable effects. A second study [22] proposed an intensive stepping verticalization protocol (1 session/day, 30 min/session, 5 x/week) with a tilt table with an integrated robotic stepping device located in the ICU. They also had 90 min of PT before and after the verticalization session, while the control group was treated with conventional in-bed physiotherapy (mobilization exercises in a supine and sitting position on the bed, without out-of-bed mobilization nor verticalization) for 60 min a day. The authors noticed a significant improvement on the Disability Rating Scale and the Coma Recovery Scale-revised in the experimental group [22]. In the last trial [23], the participants in the experimental group were treated with conventional therapy once daily (at 2 days after the patient became stable), 6 days per week, and the course of treatment was 10 days. After three treatment courses, the groups were compared. The experimental intervention included awakening therapy with transcranial direct current stimulation, hyperbaric oxygenation, sensory stimulation, fastigial nucleus stimulation, etc. The patients had a lower-limb exercise program, passive and active self-assisted exercises, and electric stimulation. Early rehabilitation therapy (experimental) decreased APACHE II (a severity score and mortality estimation in ICU), enhanced MRC (strength measures) scores, and improved the level of consciousness. Moreover, it reduced the incidence of complications (pneumonia and deep venous thrombosis) and shortened ICU or total hospital stay and mechanical ventilation time of ICU patients.

### 3.2. Studies including Prognostic Factors and/or Responses Resulting from Inpatient Rehabilitation

The second category of articles included those addressing prognostic factors and studies investigating responses to rehabilitation interventions. A first study aimed to describe the Functional Independence Measure (FIM), balance and mobility outcomes across 3 different age groups of older adults with TBI after inpatient rehabilitation but did not provide details on the PT protocol used. The authors reported a significant improvement in all outcomes (mobility with Timed Up and Go, balance with BERG Balance Scale, and walking speed with the 10-m walk test, as well as in the FIM scores [24]. A Canadian study conducted by Chan et al. [10] established the profile of the elderly with a TBI and aimed to explore the effects of an in-hospital rehabilitation program on physical function, measured at hospital discharge. They noticed important gains in the FIM scores, stating that older groups after a TBI have a rehabilitation potential similar to or better than the non-traumatic TBI [10].

In the study of Khoo and al. [25], the authors wanted to determine differences in outcomes after inpatient neurorehabilitation between younger and older adults, both assessed as having rehabilitation potential. One group received neurological specialized rehabilitation and the other group experienced conventional therapy. The authors claim that no age limit should be stipulated since older people present similar improvements when compared to the younger in terms of functional capacity (FIM + FAM-Functional Assessment Measure), reiterating that the oldest age groups can greatly benefit from specialized neurological rehabilitation as much as the youngest [25].

Another study collected clinical data on almost 1500 patients admitted to 9 inpatient rehabilitation facilities for initial rehabilitation after TBI [26]. They collected the number of patients receiving any therapy during the rehabilitation stay for six disciplines separately and by age group. Five PT activities were mostly used (across all age groups combined): gait (25% of all therapy time), therapeutic exercise (17%), standing (8%), resting (8%), and formal assessment (8%). Together these activities took up to 66% of the therapy time reported by PT, with the proportion being lower in the under-30 group and highest in the 85+ group. A separate analysis indicated that younger age groups received more hours of advanced gait training than the older. Most importantly, older patients (65 or older) had a lower brain injury severity and a shorter length of stay in acute care. During rehabilitation, they received fewer hours of therapy and treatment per day. They regained less functional ability during and after inpatient rehabilitation and had a high mortality rate [26].

Another study [27] determined the factors that influence functional intensive rehabilitation and the ones helping achieve the minimal detectable change (MDC) and the minimal clinically important difference (MCID) on the FIM-motor score. They observed that the factors with the highest probability of achieving the MCID were high motor and cognitive function at admission, and a lower number of comorbidities. For the patients who had a lower motor function, a length of stay over 10 days was a factor that helped achieve the MCID. Importantly, older age was associated with a lower FIM-M discharge score, but not the probability of achieving the MCID in the FIM-M score. Finally, another study by Lamm and al. [28] presenting the demographic characteristic of patients (n = 233,843) who were admitted between 2002 and 2016 to medical rehabilitation facilities (n = 1290) after sustaining a TBI, discusses the research and clinical implications of trends in acute-care admission for TBI in terms of age, length of stay and functional capacity [28].

Scott and al. [29] did not mention the specific PT intervention protocol but explained that the participants received traumatology care and were followed by a multidisciplinary team. They observed a decrease in the duration of the hospital and ICU stay and a decreased chance of 30-day mortality and an increased chance of having a good recovery, as per the Glasgow outcome scale [29].

The last article in this category compared a specialized rehabilitation program with a non-specialized rehabilitation program in Australia. Of those needing inpatient rehabilitation, 62% were admitted to specialist units, and the remainder were admitted to non-specialist units. Those admitted to specialist units were younger and had a lower cognitive FIM score on admission than those admitted to non-specialist units. Specialist units achieved better overall FIM score improvements from admission to discharge but at a cost of a longer length of stay. However, few older patients (19%) with brain injuries were admitted to this specialized service [30].

### 3.3. Gray Literature

Five articles from gray literature sources did not have any outcome measure as they are purely descriptive. The interventions mentioned are from experts’ opinions, recommendations, or consensus. One of the sources (Clinical Guidelines for TBI) includes 71 recommendations for an optimal TBI rehabilitation care. The recommendations suggest interdisciplinary care oriented toward the patient, a goal, or a task. It is mentioned that the duration of the stay should be determined at the start of the treatment, which should be based on other patients’ profiles with similar conditions. They suggest the rehabilitation to be initiated as soon as the state of the person allows, from a medical perspective. A post-discharge follow-up should be offered to the patient. Lastly, the family should be involved throughout the whole rehabilitation process [31]. The second article suggests that physical therapy is essential to gain movements, balance, coordination, and cognitive function after a TBI to prevent any complications. The patient should receive physiotherapy sessions during the acute stage daily, which would help with motor and cognitive improvements [32]. In the third article, they mentioned that the immediate goal of rehabilitation is to prevent secondary complications, such as articular contractures, skin damage, venous stasis, and lung function deterioration. Therefore, the therapy should include neurophysiologic therapy, rehabilitation (physical and occupational) therapy, and speech therapy [33]. In the following article [34], the authors suggest a good airway management to prevent hypoxia as well as seizure management and prevention by keeping the head inclined at 30 degrees and keeping the neck in a neutral position to reduce the risk of intracranial pressure increases. The authors state that interdisciplinary teams are essential to provide optimal care and changes trajectories of long-term recovery. The last article [35] mentions that efficient interventions include awakening, physical and functional stimulations, maintenance of mobility, normalization of muscle tone, assessing secondary complications, positioning, mobilization, airway clearance, pain management, neuromuscular facilitation, and education of the patient and their family. They also suggest a program oriented on a goal or a task and that discharge should be planned early in the rehabilitation process.

### 3.4. The Results in Brief

**Figure 2:**
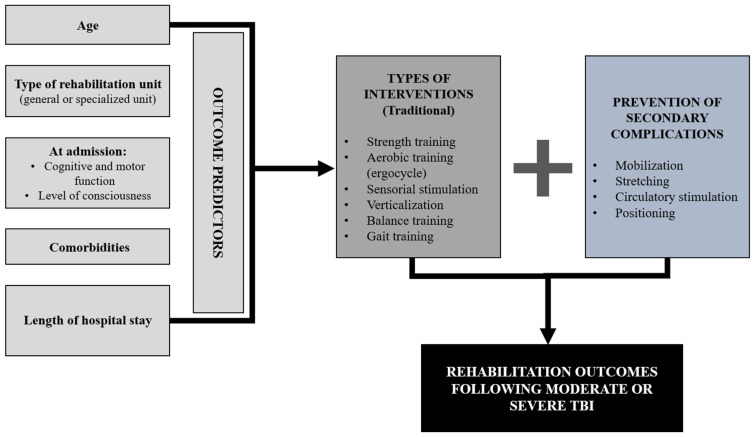
Graphic summary of findings.

To summarize, some interventions and interesting results arise from this review. Here we describe our results according to the type of intervention. A program on a stationary bicycle was only mentioned once [21]. Therapy that included strength, endurance, balance, and other components was mentioned in six studies [21,23,26,32,33,35]. Three studies have not described intervention details but one specified they were carried out in a traumatology care setting and another one mentioned a rehabilitation program [10,24,29]. Only two studies suggested a verticalization program and a walking program [22,35]. Two studies advised awakening therapy [23,35]. Three studies proposed specialized rehabilitation [25,30,33]. One article explored factors helping to achieve the clinically important improvements [27]. One study demonstrated the impact of implementing such a rehabilitation service across different age groups [28] and two studies suggested an interdisciplinary approach to rehabilitating these patients [31,34]. Speech therapy was mentioned in one study [33]. Finally, the management of airway and intracranial pressure is discussed in two articles [34,35]. [Table 2 near here]. The main findings related to prognostic factors and the main types of interventions considering studies with rehabilitation outcomes are illustrated in Figure 2.

## 4. Discussion

This review identified sixteen articles that helped us answer our objectives and highlight important needs for future research. The main finding is that there are some rehabilitation options that are specific to the older population reported in the literature but there is no standardized protocol nor high-quality evidence to make solid recommendations with regard to rehabilitation care for the elderly after a moderate or severe TBI. However, the studies demonstrated that physiotherapy has a potential positive impact on preventing secondary complications and on improving physical and functional capacities during the hospitalization period, which has a likely impact on the prevention of long-term disability and premature mortality.

### 4.1. Interventional Studies

For this category, there were three studies but only two presented intervention details that allow its reproducibility. A difference was observed in the duration of the treatment, which was between 10 days to 4 weeks, and in the duration of the sessions (30 min up to 1 h), but the number of sessions per week did not differ much (5 x/week to 6 x/week). The studies proposed exercise programs, stimulation, and stationary bicycle. The latest was not conclusive. Even with all these differences, it can be concluded that the results generated by physiotherapy are superior to not performing any type of rehabilitation during the hospitalization period. All the studies demonstrated a gain in multiple domains, such as balance, mobility, gait, and function. Many of the interventions suggested are supported by gray literature sources. In conclusion, physiotherapy should be offered to this clientele without hesitation. However, there is no consensus on the optimal protocol to be employed and more studies of better quality are needed for these interventions to reach their best potential.

### 4.2. Studies including Prognostic Factors and/or Responses Resulting from Inpatient Rehabilitation

In this section, eight articles were included, where many different factors were raised as predictors of response to treatment or as observed findings of the implementation of specific inpatient rehabilitation programs. Six studies were interested in the effect of age on the rehabilitation recovery trajectory. All concluded that being 65 years or older should not be considered as a determinant of rehabilitation delivery, nor should it be a restriction on its quantity and quality. However, age could influence the mortality rate, discharge placement decisions, hospital stay, and rehabilitation duration after a TBI [10]. Furthermore, the elderly are at greater risk of developing psychological and mental complications and having a multimorbid profile that could, in turn, affect long-term recovery from TBI [32]. Age should not, therefore, be a barrier to accessing rehabilitation services, but rather a factor to take into account when choosing an intervention, since this subpopulation has a recovery potential greater than or equal to the younger population. Although we have not found specific interventions recommended to this population, it seems plausible to infer that they would benefit from the same interventions carried out for young people. However, it remains for future studies to determine which volume (intensity, frequency, duration) and type of exercise would best benefit this population and to what extent we could expect improvements after such interventions, while considering the known prognostic factors (e.g., Glasgow score at admission and pre-admission functional an cognitive level).

Specialized rehabilitation units help reduce relocation and gain functional capacities and should be considered not only for treating seniors after a TBI but as a specialized service in all facilities where only non-specialized general units exist. Furthermore, the contribution of comorbidities and polypharmacy as predictors to response to treatment should be explored in future trials to determine their potential impact and the implications in some types of treatments, such as in the context of less conventional interventions, such as verticalization and neurofunctional stimulation approaches.

### 4.3. Strength and Limitations

Our study has several strengths. We involved PT students, with different clinical experiences at all stages of the project: conceptualization of the research question, determination of definitions and criteria, and refinement of data extraction. We used objective criteria and a pretested data extraction form to optimize reproducibility and transparency. We selected citations and completed data extraction independently and in pairs, minimizing potential error and bias. The review is, however, not without limitations. We only reviewed articles in English or French and published after 2010. Moreover, the great heterogeneity of the existing literature did not allow us to group similar interventions together and did not allow us to draw conclusions in terms of the superiority of interventions.

## 5. Conclusions

Older people show as much or greater improvement than younger adults and this is regardless of their baseline condition. The presence of comorbidities, length of hospital stay, pre-admission functional and cognitive state, and type of rehabilitation unit (specialized or not) admission play a role in determining after-TBI recovery among older adults. Some specific treatment modalities seem to induce beneficial effects, but there is not enough RCTs nor consensus, protocol, or standardized guidance that can help health professionals to make better and more enlightened decisions. Therefore, this scoping review, aiming to identify the current evidence and the knowledge and practice gaps in the context of TBI in older people, highlights the urgent need for high-quality articles to help determine what are the most promising approaches in acute and subacute settings, and how much they can induce the best possible recovery of older people who have suffered a moderate-to-severe TBI. It also suggests the need for more robust research investigating the individual effect of the disciplinary PT component of the often-proposed multidisciplinary rehabilitation programs.

## Figures and Tables

**Table 1:** Summary of the sources and characteristics of the articles included in this scoping review.

Characteristics	
1. Countries	n (%) for a total of 16
1.1 USA	5 (31)
1.2 Canada	3 (19)
1.3 United Kingdom	3 (19)
1.4 Australia	2 (13)
1.5 China	1 (6)
1.6 Poland	1 (6)
1.7 Unspecified	1 (6)
2. Journal focus	n (%) for a total of 16
2.1 Moderate to severe traumatic brain injury (TBI)	5 (31)
2.2 TBI in general	4 (25)
2.3 TBI (severe)	4 (25)
2.4 Cerebral lesions, including TBI	3 (19)
3. Journals and other sources	n (%) for a total of 16
3.1 The Journal of head trauma rehabilitation	2 (13)
3.2 Annals of Long-term Care	1 (6)
3.3 Annals of Physical and Rehabilitation Med	1 (6)
3.4 BMC Health Services Research	1 (6)
3.5 Cambridge Journal of Aging	1 (6)
3.6 Evidence-based review of moderate to severe acquired brain injury (ERABI online clinical tool)	1 (6)
3.7 Injury-International journal of the care of the Injured	1 (6)
3.8 Journal of Neurotrauma	1 (6)
3.9 Journal of Physiotherapy	1 (6)
3.10 Medical Science Monitor	1 (6)
3.11 Neurorehabilitation	1 (6)
3.12 Physiopedia	1 (6)
3.13 PLOS One	1 (6)
3.14 PM&R journal	1 (6)
3.15 Book	1 (6)
4. Primary journal audience	n (%) for a total of 16
4.1 Medical	5 (31)
4.2 Rehabilitation	5 (31)
4.3 Physiotherapy	2 (13)
4.4 Geriatric	1 (6)
4.5 Science in general	1 (6)
5. Publication type	n (%) for a total of 16
5.1 Retrospective study	8 (50)
5.2 Randomized clinical trial	4 (25)
5.3 Book	2 (13)
5.4 Description of models of practice	1 (6)
5.5 Reports	1 (6)

**Table 2:** Summary description of the studies included in the review.

Author and Year of Publication	Design	Population	Interventions	Main Findings
Interventional studies				
De Sousa et al., 2016 [21]	RCT	≈60 y Non-progressive acquired brain injury, including traumatic brain injury (TBI) (n = 40)	Functional electrical stimulation (FES) on ergocycle (5 times/week, 4 weeks, 17–32 min/session). Interdisciplinary teamwork.	Mobility, assessed using 3 items of the Functional Independence Measure (bed-chair transfer, walking and stairs) and knee extensor strength (assessed with a hand-held dynamometer) did not change after intervention. Possible improvement in leg muscle strength (assessed by manual muscle testing): difference of 3, 0/20 points (IC at 95 % 1.3 to 4.8) after intervention.
Frazzitta et al., 2016 [22]	Randomized pilot study (Parallel group)	≥18 y Severe TBI (n = 40)	Verticalization (daily sessions of 30 min, 15 sessions/patient) and convention in-bed physical therapy (30 min), 5 times/week for 3 weeks.	77% of the patient completed the study without adverse effects. The ICU length of stay was longer in the verticalization group (38.8 ± 15.7 vs. 25.1 ± 11.2 days) while the total length of stay was not significantly different (ICU + Neurorehabilitation). All outcome measures significantly improved in both groups. Higher improvement in the experimental group for Coma Recovery Scale revised (CRSr) and Disability Rating Scale (DRS).
Pang et al., 2019 [23]	RCT	18 to 80 y Cerebral injuries including TBI (n = 42)	Conventional therapy. Awakening therapy with transcranial direct current stimulation. Hyperbaric oxygenation. Sensory stimulation. Fastigial nucleus stimulation. Lower limb exercise program. Passive and active self-assisted exercises. Electric stimulation.	In the early rehabilitation group, the acute physiology and chronic health evaluation score (APACHE II) decreased. Enhanced MRC scale scores. Improved consciousness of ICU patients. Reduced the incidence of complications and shortened ICU stay.
Studies addressing prognostic factors and/or responses resulting from inpatient rehabilitation				
Perry et al., 2019 [24]	Retrospective case series	≥65 y TBI (n = 100)	Inpatient rehabilitation.	Significant improvement in mobility, balance and gait speed (Timed up and go, Berg Balance Scale, and Gait Speed). No differences in the amount of change by age. Improvements in Functional Independence Measure (FIM) Walk and Transfer scores in all age groups.
Chan et al., 2013 [10]	Retrospective cohort study	≥65 y (n = 1214 TBI and 1530 non-traumatic TBI from 2003 to 2009)	In-patient rehabilitation. with a previous acute care admission.	While both TBI and nTBI patients made significant gains in FIM from admission to discharge, a comparison of absolute change in FIM among these two groups across all referral discharge destinations revealed that the gains made were not significantly different. Even though younger adults with TBI and nTBI who were discharged home from in-patient rehabilitation had significantly higher FIM scores at admission and at discharge compared to older adults discharged home, the absolute change in FIM score was not significantly different, suggesting that these patients make similar gains in rehabilitation.
Dijkers et al., 2013 [26]	Retrospective study	≥14 y divided 6 groups (<30, 30–44, 45–64, 65–74, 75–84, ≥85) TBI severe enough to warrant inpatient rehabilitation (n = 1419, 24% of patients ≥ 65 y, from 9 inpatient facilities)	Interdisciplinary teamwork: psychology, physical therapy, occupational therapy, therapeutic recreation, and speech and language pathology. Physical therapy interventions: therapeutic exercise, bed mobility, equipment management sitting-standing-transfers, wheelchair mobility, gait training, resting, patient home assessment, etc.	The five activities most used (across all age groups combined) in PT are Gait (25% of all therapy time), Therapeutic exercise (17%), Standing (8%), Resting (8%), and Formal assessment (8%). The mean minutes per week is higher for the older age groups for Gait and Resting; on the other hand, younger patients get more minutes per week of Therapeutic exercise. For all disciplines combined, the overall difference in hours of received intervention is statistically significant (under-30 group receives more treatment than the 45–64 and older groups). FIM gains during rehabilitation, and after rehabilitation, are the largest for the younger age groups. The cognitive FIM scores were higher in the youngest groups at 3 and 9 months. For both Motor and Cognitive FIM the time of greatest change in FIM scores is during the rehabilitation stay and the first 3 months post discharge. A total of>90% of the young patient were discharged home and <65% of the elderly were discharged home. The percent living at home increases by 9 months post discharge to 74% in the oldest group, with a statistically significant difference between age groups. By the time of rehabilitation discharge, there was less improvement in both Motor and Cognitive FIM scores in the oldest age groups, which may be explained by the shorter length of stay for these groups. The patients admitted for TBI were older, had a less severe injury but more functional dependence pre-admission and comorbidities.
Evans et al., 2021 [27]	Retrospective cohort study	≥66 y Mild to severe TBI (n = 1178 from 2011 to 2015)	Inpatient rehabilitation.	Among older adults with TBI, significant impairments in cognitive and motor function are associated with increased risk of an unsuccessful inpatient rehabilitation facility admission. A total of 84% of patients achieved the minimal detectable change and 68% achieved the minimal clinically important difference (MCID) for the FIM-M score. The factors associated with a better chance of achieving the MCID was higher motor and cognitive function at admission, less comorbidities, and length of stay >10 days but only for patients with a low baseline function. Higher age was associated with a lower FIM-M score at discharge, but not with the chances of achieving the MCID.
Khoo et al., 2020 [25]	Retrospective study	≥65 and <65 y. Brain and peripheral injuries (TBI 70%, n = 429/616 from 2011 to 2016)	Specialized neurorehabilitation. Interdisciplinary teamwork.	Improvement of the FIM and FAM score for the elderly. UK Functional Independence Measure + Functional Assessment Measure (FIM + FAM) scores at admission were significantly lower in the older than younger group. Average LOS did not differ. Both UK FIM + FAM change and efficiency (adjusted by LOS) were significantly higher for older than younger patients. A total of 6% younger and 11% elderly needed a relocation to long-term care. Older age was associated with the need for LTC placement.
Lamm et al., 2019 [28]	Retrospective study	<55, 55–64, > 64 y. TBI from 2002 to 2016 (n = 233,843 from 1290 facilities in USA)	Neurorehabilitation.	Compared to their previous work, the mean age increased from 54 to 65, rehabilitation length of stay decreased from 19 to 14 days, and the FMI score from 56.9 to 54.5. Patients admitted to medical rehabilitation facilities after a TBI are significantly older, less functional on admission, staying at rehabilitation facilities for shorter periods of time, but with similar functional status at the time of discharge.
Scott et al., 2021 [29]	Retrospective study	≥18 y (mean age: 57) Patients admitted to a Major Trauma Center including n = 1970/6484, 28% of head trauma from 2012 to 2018)	Interdisciplinary teamwork: consultant allied health professional, rehabilitation leads, occupational therapy, physiotherapy, speech and language therapy, dietetics, psychology, social work and generic rehabilitation support.	Introducing of a rehabilitation service into the major trauma center was associated with a reduction in hospital length of stay of 2.56 days, a reduction in ICU length of stay of 0.94 days, and almost two times higher relative chance (1.94) of a Glasgow outcome score of Good Recovery. After the introduction of the service, patients were significantly older and presented more often with higher injury severity scores. A 31% reduction in 30-day mortality post-implementation of the service.
Wu et al., 2018 [30]	Retrospective study	>18 y, 16% patients ≥65 y, mean age 42 y) TBI in general (n = 268/667, 40% TBI from 2009 to 2012)	Trauma care units (specialist and non-specialist inpatient rehabilitation units). Interdisciplinary teamwork.	Of those who were >65 y, only 19% were admitted to a specialist unit. Of the patients accepted by specialist units, only 5% were >65 y. Those treated in non-specialist units were usually older, had less severe injuries and were functionally and cognitively less impaired at admission to rehabilitation compared with those admitted to specialist units. The non-specialist units achieved comparable discharge FIM scores to those of specialist units. Specialist units achieved significantly greater FIM gain compared with non-specialist units but at a cost of longer length of stay (lower FIM efficiency change. The trend is that younger and more severely injured patients are being managed in specialist units with the older, less cognitively impaired patients managed in non-specialist units).
Gray literature				
Bayley et al., 2018 [31]	N/A	Moderate-to-severe TBI	N/A	The final recommendation set were divided in 2 sections: Section I: Components of the Optimal TBI Rehabilitation System (71 recommendations) and Section II: Assessment and Rehabilitation of Brain Injury Sequelae (195 recommendations). The recommendations address top priorities for the TBI rehabilitation system: (1) intensity/frequency of interventions; (2) rehabilitation models; (3) duration of interventions; and (4) continuity-of-care mechanisms. Key sequelae addressed (1) behavioral disorders; (2) cognitive dysfunction; (3) fatigue and sleep disturbances; and (4) mental health. From the 71 recommendations, some are applicable to physiotherapy: Interdisciplinary care oriented to the patient; Initiate rehabilitation as soon as the condition of the person with TBI allows; A target length of stay should be established as soon as possible after admission to inpatient rehabilitation to ensure consistency of care and to facilitate discharge planning and community integration. The target length of stay should be established on the basis of individuals with similar functional status and availability of resources and take into account other factors such as the Glasgow Coma Score in the first days after injury, intracranial surgery, the degree of initial disability, and the person’s age.
Meyer et al., 2018 [32]	N/A	≥65 y and adults Moderate-to-severe TBI	Physiotherapy every day during the acute phase. Head elevation at 30° (intracranial pressure). Electrical stimulation. Sensory stimulation (auditory, tactile, multimodal). Verticalization.	4% of elderly with an initial Glasgow Coma scale <8 have a good recovery. Decreased mental and physical health observed in the elderly after a TBI. 5–20% have a moderate recovery 1year post-TBI. A low Glasgow Coma scale predicts poor long-term results. The elderly with a TBI and neurodegenerative comorbidities will experience a more pronounced decline. Geriatric Rehabilitation Program helps the elderly be more independent with shorter stay and better improvements.
Physiopedia 2022 (Ziemer, Anna-original editor) [35]	N/A	Moderate-to-severe TBI	Awakening stimulation. Functional and physical stimulation. Mobility stimulation. Muscle tone normalization. Prevent or reduce secondary complication. Early mobilization. Airway clearance. Pain management. Manual therapy. Family and Caregiver’s Education. Education on equipment use. Neuromuscular stimulation. Positioning. Rising safety awareness. Balance and Postural control training. Verticalization.	The interventions described are for adults and older people after a TBI at each stage of the rehabilitation.
Stippler et al., 2012 [33]	N/A	≥65 y, TBI	Prevent complications caused by immobilization.	Despite the general improvement in outcomes after TBI over the past 50 years, the outcome for elderly patients remains poor. During the past 10 years, the mortality linked with TBI has increased because of the aging of the population. Rehabilitation should be initiated shortly after hospital admission, even while a patient is still in an ICU setting. The immediate goal of rehabilitation is to prevent complications associated with a prolonged period of immobilization, such as joint contracture, skin breakdown, venous stasis, and pulmonary compromise. Rehabilitation strategies include neuropsychologic, physical, occupational, and speech therapies
Yee et al., 2021 [34]	N/A	≥65 y, severe TBI	Airway management. Seizure management. Cardiovascular management. Inclined the head at 30º and keep the neck neutral. Fall prevention. Interdisciplinary teamwork.	The elderly (60–99 y) with a severe TBI (GCS < 9) have a 80% chance of dying or having long-term disability. Survivors of TBI may suffer from chronic neurological complications. Seizures are a common long-term complication as well as varying degrees of cognitive impairment. Focal weakness or sensory deficits may also occur.

N/A = Not applicable.

## Data Availability

Not applicable.

## Appendix A

**Table A1:** Downs and Black Checklist for Measuring Study Quality: Summary of included studies.

Downs and Black Checklist												
Study Design		Interventional Studies (Randomized and Non-Randomized Trials)			Studies Addressing Prognostic Factors and/or Responses Resulting from Inpatient Rehabilitation (Retrospective Studies)							
Articles (Authors, Year of Publication)		Frazzitta et al., 2016	De Sousa et al., 2016	Pang et al., 2019	Perry et al., 2019	Chan et al., 2013	Khoo et al., 2019	Dijkers et al., 2013	Evans et al., 2012	Lamm et al., 2019	Scott et al., 2021	Wu et al., 2018
**Reporting**	1. Is the hypothesis/aim/objective of the study clearly described?	1	1	1	0	1	1	0	1	1	1	1
	2. Are the main outcomes to be measured clearly described in the Introduction or Methods section?	1	1	1	0	1	1	1	1	1	1	1
	3. Are the characteristics of the patients included in the study clearly described?	1	1	1	1	1	1	1	1	1	0	1
	4. Are the interventions of interest clearly described?	1	1	1	1	0	1	1	0	0	0	0
	5. Are the distributions of principal confounders in each group of subjects to be compared clearly described?	1	2	2	0	0	0	2	0	2	2	1
	6. Are the main findings of the study clearly described?	1	1	1	1	1	1	1	0	1	0	1
	7. Does the study provide estimates of the random variability in the data for the main outcomes?	1	1	1	1	1	1	1	1	1	0	1
	8. Have all important adverse events that may be a consequence of the intervention been reported?	1	0	1	N/A	N/A	N/A	N/A	N/A	N/A	N/A	N/A
	9. Have the characteristics of patients lost to follow-up been described?	1	1	1	N/A	N/A	N/A	N/A	N/A	N/A	N/A	N/A
	10. Have actual probability values been reported (e.g., 0.035 rather than < 0.05) for the main outcomes except where the probability value is less than 0.001?	1	0	0	1	1	1	1	1	1	0	0
**External validity**	11. Were the subjects asked to participate in the study representative of the entire population from which they were recruited?	1	0	0	1	1	1	1	0	1	1	1
	12. Were those subjects who were prepared to participate representative of the entire population from which they were recruited?	1	0	0	N/A	N/A	N/A	N/A	N/A	N/A	N/A	N/A
	13. Were the staff, places, and facilities where the patients were treated, representative of the treatment the majority of the patients receive?	1	1	0	1	0	0	1	1	1	1	1
**Internal validity-bias**	14. Was an attempt made to blind study subjects to the intervention they have received?	1	0	0	N/A	N/A	N/A	N/A	N/A	N/A	N/A	N/A
	15. Was an attempt made to blind those measuring the main outcomes of the intervention?	1	1	0	N/A	N/A	N/A	N/A	N/A	N/A	N/A	N/A
	16. If any of the results of the study were based on “data dredging”, was this made clear?	1	0	1	1	0	1	1	0	0	0	1
	17. In trials and cohort studies, do the analyses adjust for different lengths of follow-up of patients, or in case-control studies, is the time period between the intervention and outcomes the same for cases and controls?	0	1	0	1	1	1	1	1	0	1	1
	18. Were the statistical tests used to assess the main outcomes appropriate?	1	1	1	1	1	1	1	1	1	1	1
	19. Was compliance with the intervention/s reliable?	1	1	1	N/A	N/A	N/A	N/A	N/A	N/A	N/A	N/A
	20. Were the main outcome measures used accurate (valid and reliable)?	1	1	1	1	1	1	1	1	1	1	1
**Internal validity-confounding (selection bias)**	21. Were the patients in different intervention groups (trials and cohort studies) or were the cases and controls (case-control studies) recruited from the same population?	1	1	1	1	1	1	0	1	0	0	1
	22. Were study subjects in different intervention groups (trials and cohort studies) or were the cases and controls (case-control studies) recruited over the same period of time?	1	1	1	1	1	1	0	1	1	0	1
	23. Were study subjects randomized to intervention groups?	1	1	1	N/A	N/A	N/A	N/A	N/A	N/A	N/A	N/A
	24. Was the randomized intervention assignment concealed from both patients and health care staff until recruitment was complete and irrevocable?	1	1	0	N/A	N/A	N/A	N/A	N/A	N/A	N/A	N/A
	25. Was there adequate adjustment for confounding in the analyses from which the main findings were drawn?	0	1	0	1	1	1	1	0	0	0	0
	26. Were losses of patients to follow-up taken into account?	1	1	1	N/A	N/A	N/A	N/A	N/A	N/A	N/A	N/A
**Power**	27. Did the study have sufficient power to detect a clinically important effect where the probability value for a difference being due to chance is less than 5%?	0	0	1	1	1	1	1	0	0	0	0
**Total Score**		**24/28 (86%)**	**21/28 (75%)**	**19/28 (68%)**	**15/19 (79%)**	**14/19 (74%)**	**16/19 (84%)**	**16/19 (84%)**	**11/19 (58%)**	**13/19 (68%)**	**9/19** **(47%)**	**14/19 (74%)**

N/A = Not applicable.

## Appendix B

**Table A2:** Authority, Accuracy, Coverage, Objectivity, Date, Significance (ACCODS) Checklist for Measuring Gray Literature Sources Quality: Summary of included reports.

Domain	Items assessed (When Applicable)	1 INESS, 2018	2 ERABI, 2018	3 Stippler et al., 2012	4 Yee et al., 2021	5 Physiopedia 2022
**Authority**	**1. Identifying who is responsible for the intellectual content.**					
	**1. Individual author**					
	1.1 Associated with a reputable organization?	Yes	Yes	Yes	Yes	Yes
	1.2 Professional qualifications or considerable experience?	Yes	Yes	Yes	Yes	Yes
	1.3 Produced/published other work (grey/black) in the field?	Yes	Yes	Yes	Yes	Yes
	1.4 Recognized expert, identified in other sources?	Yes	Yes	Yes	Yes	N/A
	1.5 Cited by others? (used Google Scholar as a quick check)	Yes	Yes	Yes	Yes	No
	1.6 Higher degree student under “expert” supervision?	No	No	Yes	No	No
	**2. Organisation or group**					
	2.1 Is the organization reputable? (e.g., W.H.O)	Yes	Yes	No	Yes	No
	2.2 Is the organization an authority in the field?	Yes	No	No	No	No
	**3. In all cases**					
	3.1 Does the item have a detailed reference list or bibliography?	Yes	Yes	Yes	Yes	Yes
	**4. Comments**					
**Accuracy**	Does the item have a clearly stated aim or brief?	Yes	Yes	Yes	No	No
	Is so, is it met?	Yes	Yes	Yes	N/A	N/A
	Does it have a stated methodology?	Yes	No	No	N/A	No
	If so, is it adhered to?	Yes	N/A	N/A	N/A	N/A
	Has it been peer-reviewed?	Yes	No	No	N/A	No
	Has it been edited by a reputable authority?	Yes	Yes	No	Yes	No
	Supported by authoritative, documented references or credible sources?	Yes	Yes	Yes	Yes	Yes
	Is it representative of work in the field?	Yes	Yes	Yes	Yes	Yes
	If No, is it a valid counterbalance?	N/A	N/A	Yes	N/A	N/A
	Is any data collection explicit and appropriate for research?	Yes	Yes	Yes	N/A	N/A
	If item is secondary material (e.g., a policy brief of a technical report refers to the original.	N/A	N/A	N/A	N/A	N/A
	Is it an accurate, unbiased interpretation or analysis?	Yes	Yes	No	N/A	N/A
	**Comments:**					
**Coverage**	All items have parameters which define their content coverage. These limits might mean that work refers to a particular population group, or that it excluded certain types of publication. A report could be designed to answer a particular question or be based on statistics from a particular survey.					
	Are any limits clearly stated?	Yes	No	No	N/A	N/A
**Objectivity**	Is it important to identify bias, particularly if it is unstated or unacknowledged.					
	Opinion, expert or otherwise, is still opinion: is the author’s standpoint clear?	Yes	Yes	No	Yes	No
	Does the work seem to be balanced in presentation?	Yes	Yes	No	N/A	N/A
	**Comments:**					
**Date**	For the item to inform your research, it needs to have a date that confirms relevance					
	Does the item have a clearly stated date related to content? No easily discernible date is a strong concern.	Yes	No	Yes	Yes	No
	If no date is given, but can be closely ascertained, is there a valid reason for its absence?	N/A	No	Yes	N/A	N/A
	Check the bibliography: have key contemporary material been included?	Yes	Yes	Yes	Yes	Yes
	**Comments:**					
**Significance**	This is a value judgment of the item, in the context of relevant research area					
	Is the item meaningful? (This incorporates feasibility, utility, and relevance)	Yes	Yes	Yes	Yes	Yes
	Does it add context?	Yes	Yes	Yes	N/A	N/A
	Does it enrich or add something unique to the research?	Yes	Yes	No	N/A	N/A
	Does it strengthen or refute a current position?	Yes	Yes	No	No	No
	Would the research area be lesser without it?	Yes	Yes	No	No	No
	Is it integral, representative, typical?	Yes	Yes	No	N/A	N/A
	Does it have impact? (In the sense of influencing the work or behavior of others)	Yes	Yes	No	Yes	Yes
**Total results: /34**		**30/32 (94%)**	**24/31 (77%)**	**18/32 (56%)**	**14/18 (78%)**	**9/19 (47%)**

N/A = Not applicable.
